# Prevalence of Multiple Chronic Conditions Among US Adults, 2018

**DOI:** 10.5888/pcd17.200130

**Published:** 2020-09-17

**Authors:** Peter Boersma, Lindsey I. Black, Brian W. Ward

**Affiliations:** 1 Division of Health Interview Statistics, National Center for Health Statistics, Hyattsville, Maryland; 2 Division of Health Care Statistics, National Center for Health Statistics, Hyattsville, Maryland

## Abstract

This analysis provides prevalence estimates of diagnosed single and multiple (≥2) chronic conditions among the noninstitutionalized, civilian US adult population. Data from the 2018 National Health Interview Survey (NHIS) were used to estimate percentages for US adults by selected demographic characteristics. More than half (51.8%) of adults had at least 1 of 10 selected diagnosed chronic conditions (arthritis, cancer, chronic obstructive pulmonary disease, coronary heart disease, current asthma, diabetes, hepatitis, hypertension, stroke, and weak or failing kidneys), and 27.2% of US adults had multiple chronic conditions.

**Summary**

**What is already known on this topic?**

Research has shown increases in the overall percentage of US adults with multiple chronic conditions, as well as variation by population subgroups.

**What is added by this report?**

In 2018, 51.8% of US adults had at least 1 chronic condition, and 27.2% had multiple chronic conditions. Prevalence was highest among women, non-Hispanic white adults, adults aged 65 or older, and those living in rural areas.

**What are the implications for public health practice?**

These results may inform efforts to track and monitor multiple chronic conditions among the US population.

## Objective

In 2012, 25.5% of US adults had multiple (≥2) diagnosed chronic conditions among 10 different conditions: arthritis, cancer, chronic obstructive pulmonary disease (COPD), coronary heart disease, current asthma, diabetes, hepatitis, hypertension, stroke, and weak or failing kidneys (1,2). Compared with adults without chronic conditions, adults with multiple chronic conditions have worse health-related quality of life, higher health care costs, and increased risk of death (3–5). Using 2018 National Health Interview Survey (NHIS) data, we provide estimates of the prevalence of single and multiple chronic conditions among US adults.

## Methods

The NHIS is a cross-sectional, in-person, nationally representative health survey of the US civilian noninstitutionalized population conducted by the National Center for Health Statistics (NCHS). Data on chronic conditions are part of the sample adult component; the sample adult is randomly selected from all adults in the family. This analysis includes 25,417 sample adults from the 2018 NHIS (final response rate, 53.1%) (6).

The chronic conditions included in this study were 10 conditions from a list of 20 identified by the US Department of Health and Human Services to foster a more consistent and standardized approach to measuring the occurrence of chronic conditions (2). Adults were asked if they had ever been told by a doctor or health care provider that they had hypertension, coronary heart disease, stroke, diabetes, cancer, arthritis, or hepatitis; had experienced weak or failing kidneys during the past 12 months; currently had asthma; or had COPD (ie, ever had emphysema, ever had COPD, and/or had chronic bronchitis in the past 12 months) (1,7). Based on their responses, adults were identified as having 0, 1, or ≥2 conditions. Estimates were generated using SUDAAN software version 11.0.1 (RTI International) to account for the complex sample design of the NHIS; 95% confidence intervals were generated by using the Korn-Graubard method. All prevalence estimates met NCHS reliability standards (8). Urbanicity of residence was dichotomized based on the 2013 NCHS urban–rural classification scheme for counties (9). Two-tailed significance tests were performed to determine whether significant differences exist for percentages of multiple chronic conditions by demographic subgroup. Significance was set at *P* < .05.

## Results

In 2018, 51.8% (129 million) of civilian, noninstitutionalized adults had been diagnosed with at least 1 of 10 selected chronic conditions. More specifically, 24.6% (61 million) of adults had 1 chronic condition, and 27.2% (68 million) had ≥2 chronic conditions (Table).

**Table:** Percentage and Number of US Adults Aged 18 Years or Older With Chronic Conditions,^a^ by Select Characteristics, United States, 2018

Characteristic	0 Chronic Conditions		1 Chronic Condition		≥2 Chronic Conditions	
	% Population^b^ (95% CI)	N	% Population^b^ (95% CI)	N	% Population^b^ (95% CI)	N
**Total^c^**	48.2 (47.3–49.1)	120,230	24.6 (23.9–25.3)	61,371	27.2 (26.5–27.9)	67,854
**Sex^d^**						
Male	49.8 (48.5–51.0)	59,921	24.4 (23.4–25.4)	29,346	25.9 (24.9–26.9)	31,175
Female	46.7 (45.6–47.9)	60,309	24.8 (23.9–25.7)	32,025	28.4 (27.5–29.4)	36,679
**Race/ethnicity^e^**						
Non-Hispanic White	43.8 (42.7–44.8)	68,839	25.6 (24.8–26.4)	40,248	30.6 (29.7–31.6)	48,202
Non-Hispanic Black	47.6 (45.0–50.2)	13,845	25.4 (23.3–27.6)	7,390	27.0 (25.0–29.1)	7,855
Non-Hispanic Asian	62.0 (58.6–65.2)	9,400	21.6 (19.0–24.4)	3,280	16.4 (14.0–19.0)	2,486
Hispanic	61.5 (59.3–63.5)	25,042	20.8 (19.1–22.5)	8,478	17.7 (16.2–19.3)	7,230
**Age, y^d^**						
18–44	72.6 (71.4–73.7)	83,444	20.7 (19.7–21.8)	23,841	6.7 (6.1–7.3)	7,723
45–64	36.6 (35.3–37.9)	30,404	30.4 (29.2–31.6)	25,250	33.0 (31.7–34.3)	27,383
≥65	12.4 (11.5–13.3)	6,382	23.9 (22.7–25.1)	12,280	63.7 (62.3–65.1)	32,748
**Health insurance coverage^f^**						
**Age 18–64 y^d^**						
Private	58.6 (57.5–59.8)	80,085	25.7 (24.7–26.7)	35,065	15.7 (14.9–16.5)	21,418
Public	48.9 (46.3–51.6)	12,192	23.4 (21.4–25.5)	5,830	27.6 (25.5–29.9)	6,886
Uninsured	66.8 (64.3–69.3)	17,059	21.6 (19.4–23.9)	5,511	11.6 (10.1–13.2)	2,995
**Age ≥65 y^g^**						
Private	12.4 (11.1–13.8)	2,633	24.4 (22.6–26.3)	5,190	63.2 (61.2–65.3)	13,451
Dual eligible	6.8 (4.6–9.6)	239	16.4 (13.0–20.1)	577	76.9 (72.5–80.8)	2,713
Medicare Advantage	11.9 (10.2–13.6)	1,556	25.2 (22.9–27.6)	3,300	63.0 (60.3–65.6)	8,257
Medicare only excluding Medicare Advantage	16.1 (13.6–18.8)	1,280	25.4 (22.2–28.9)	2,020	58.5 (54.8–62.1)	4,645
**Location of residence^d^**						
Urban	49.5 (48.5–50.5)	107,383	24.5 (23.7–25.2)	53,125	26.1 (25.3–26.9)	56,577
Rural	39.7 (37.3–42.1)	12,847	25.5 (23.8–27.2)	8,246	34.8 (32.8–37.0)	11,277

a Chronic conditions measured were arthritis, cancer, chronic obstructive pulmonary disease, coronary heart disease, current asthma, diabetes, hepatitis, hypertension, stroke, and weak/failing kidneys.

b Population in 1,000s.

c Total 2018 US adult population: 249 million people. Numbers may not sum to group totals because of rounding.

d Difference in estimates of multiple chronic conditions (≥2 conditions) between all subcategories was significant at *P* < .001.

e With the exception of Hispanic vs non–Hispanic Asian adults (*P* = .37), all other differences in estimates of multiple chronic conditions among racial/ethnic groups were significant (all *P* < .001 except for between non-Hispanic White and non-Hispanic Black adults [*P* = .002]).

f Health insurance coverage was based on a hierarchy of mutually exclusive categories (6).

g With the exception of private vs Medicare Advantage (*P* = .88), all other differences in estimates of multiple chronic conditions among adults aged ≥65 by insurance status were significant (all *P* < .001 except for between private and Medicare [*P* = .02] and between Medicare Advantage and Medicare [*P* = .04]).

Prevalence of multiple chronic conditions differed by population subgroups. Prevalence was higher among women (28.4%) than men (25.9%) and increased with advancing age. Prevalence of multiple chronic conditions was highest among non-Hispanic white adults (30.6%) and lowest among non-Hispanic Asian (16.4%) and Hispanic adults (17.7%). Among adults aged 18–64 years, the prevalence of multiple chronic conditions was higher among adults on public insurance (27.6%) than either adults on private insurance (15.7%) or uninsured adults (11.6%), and the prevalence differed significantly across all insurance subgroups. Among adults aged 65 or older, the prevalence of multiple chronic conditions was highest among adults with both Medicare and Medicaid (“Dual eligible”) (76.9%), lowest among adults on Medicare only (excluding Medicare Advantage) (58.5%), and differed significantly across all insurance subgroups with the exception of those with private (63.2%) and Medicare Advantage coverage (63.0%). Examination by urbanicity indicated that those living in rural areas (34.8%) had a higher prevalence of multiple chronic conditions compared with those living in urban areas (26.1%).

## Discussion

In 2018, just over a quarter (27.2%) of US adults had multiple chronic conditions. This finding is consistent with previous research that found the prevalence to be 25.5% in 2012, 26.0% in 2010, and 21.8% in 2001 (1,10). In 2018, prevalence of multiple chronic conditions was higher among women, non-Hispanic white adults, older adults, adults aged 18–64 on Medicaid, dual-eligible adults (Medicare and Medicaid), and adults in rural areas. These disparities were similar to those identified in previous research, which found a higher prevalence among women, older adults, and adults on public insurance but did not examine prevalence by location of residence or separate insurance status by age (1,10).

This study has several limitations. First, although NHIS data are nationally representative of the civilian, noninstitutionalized population, the exclusion of institutionalized adults, particularly those in long-term care settings, may lead to underreporting of the prevalence of chronic conditions. Second, NHIS captures only 10 of the 20 chronic conditions outlined by the US Department of Health and Human Services (2). However, it is important to note that this is the first report on overall prevalence to use the expanded definition of COPD, which now includes a question that asks explicitly about diagnosed COPD in addition to questions that ask about diagnosed emphysema and chronic bronchitis (7). Third, data are based on self-report of diagnosed conditions, which may result in recall bias. Lastly, NHIS collects data only on diagnosed conditions, so undiagnosed conditions are not recorded.

We found that nearly 30% of the US adult population has multiple chronic conditions. This work builds on previous research and provides current estimates of the prevalence using a nationally representative sample. These findings may allow for an expanded understanding of the epidemiology of multiple chronic conditions by providing estimates of overall burden and identifying high-risk subgroups.
